# Guidelines for writing a commentary

**DOI:** 10.3402/qhw.v11.31390

**Published:** 2016-03-11

**Authors:** Carina Berterö

**Affiliations:** Editor-in-Chief

A commentary is a comment on a newly published article. A commentary may be invited by the chief editor or spontaneously submitted. Commentaries in *International Journal of Qualitative Studies on Health and Well-being* are peer reviewed. We now welcome commentaries!

## What is a commentary?

The goal of publishing commentaries is to advance the research field by providing a forum for varying perspectives on a certain topic under consideration in the journal. The author of a commentary probably has in-depth knowledge of the topic and is eager to present a new and/or unique viewpoint on existing problems, fundamental concepts, or prevalent notions, or wants to discuss the implications of a newly implemented innovation. A commentary may also draw attention to current advances and speculate on future directions of a certain topic, and may include original data as well as state a personal opinion. While a commentary may be critical of an article published in the journal, it is important to maintain a respectful tone that is critical of ideas or conclusions but not of authors.

In summary, a commentary may be:

1. A critical challenge to one or more aspects of the focal article, arguing for a position other than that taken in the focal article.
2. An elaboration or extension of the position taken in the focal article, basically sympathetic to the position taken in the focal article but pushing the argument further.
3. An application of a theoretical or methodological perspective that sheds light on the issues addressed in the focal article.
4. A reflection on the writer's experiences in applying the issues addressed in the focal article, in particular health and well-being settings.
5. A comment on the applicability of the issues raised in the focal article to other settings, or to other cultures.

## How to write a commentary

Commentaries in *International Journal of Qualitative Studies on Health and Well-being* should not exceed 10 manuscript pages. A tightly argued four- to six-page commentary is likely to be better received than a meandering 10-page ditto. Use these simple guidelines:

- Do not summarize the focal article; just give the reference. Assume the reader has just read it. Move directly to identifying the key issues you want to raise.
- Do not include general praise for the focal article.
- Use only essential citations. For commentary purposes, cite only works absolutely essential to support your point.
- Use a short title that emphasizes your key message. (It should be clear in context that all commentaries are a reaction to a particular paper).
- Do not include an abstract.
- Make clear your take-home message.
- Make sure there is full author information (name, affiliation, address, phone, email) for all authors. Authors must be individuals.

## Review process

Commentaries will be peer reviewed and most likely accepted if they are in line with the definitions and guidelines outlined. A small set of reviewers will read and evaluate all commentaries as they need to compare commentaries for issues of redundancy and to make evaluations of relative merit.

## Queries for the editor

Authors should feel free to correspond with the chief editor prior to submitting a commentary if there are questions about any aspect of the evaluation and publication process. Authors may prepare a brief outline of the key points they desire to present in the commentary and send it to the chief editor.

## Does it cost anything to submit a commentary?

Spontaneously submitted commentaries incur a cost of €65 per typeset page. The author will be invoiced once the commentary has been accepted for publication.

We hope you will send us a commentary whenever you think there is a need to broaden the perspectives on health and well-being presented in our journal.

*Carina Berterö* Editor-in-Chief

